# Supplementary figures and images for: Harnessing Genomics of Diaporthe amygdali for Improved Control of Peach Twig Canker and Shoot Blight (TCSB)
Source: Plants (Basel). 2025 Sep 24;14(19):2960. doi: 10.3390/plants14192960 (PMC12526002; doi:10.3390/plants14192960)

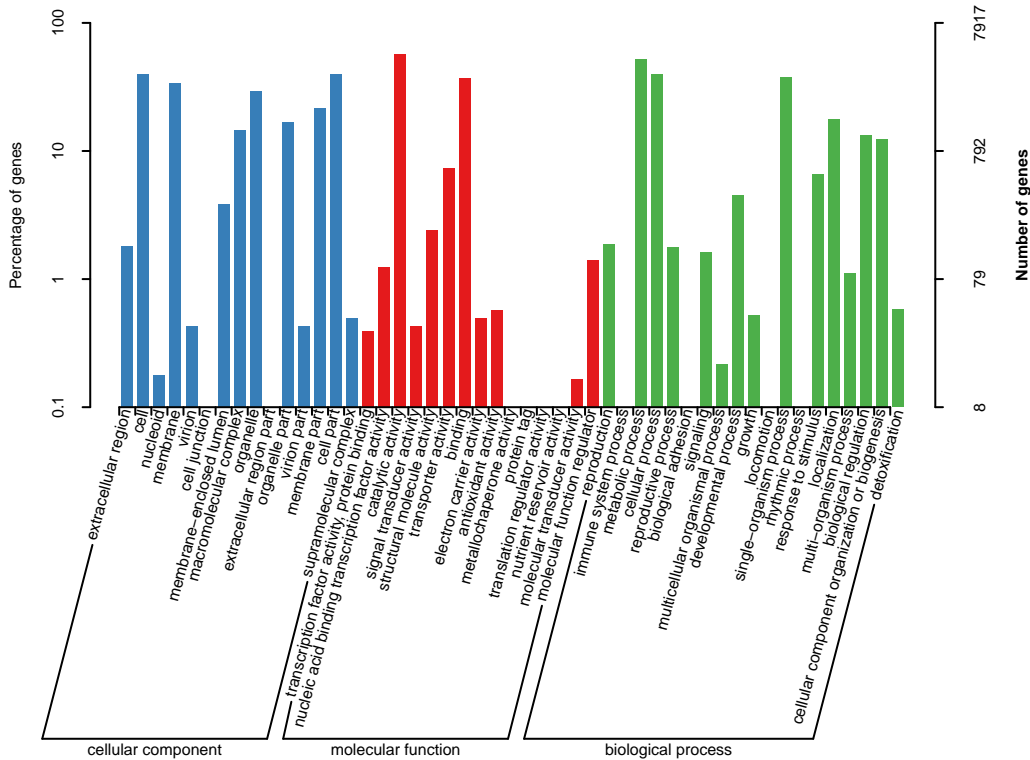

Supplement: Supplementary file 1 [file plants-14-02960-s001.zip › Figure_S1.pdf]

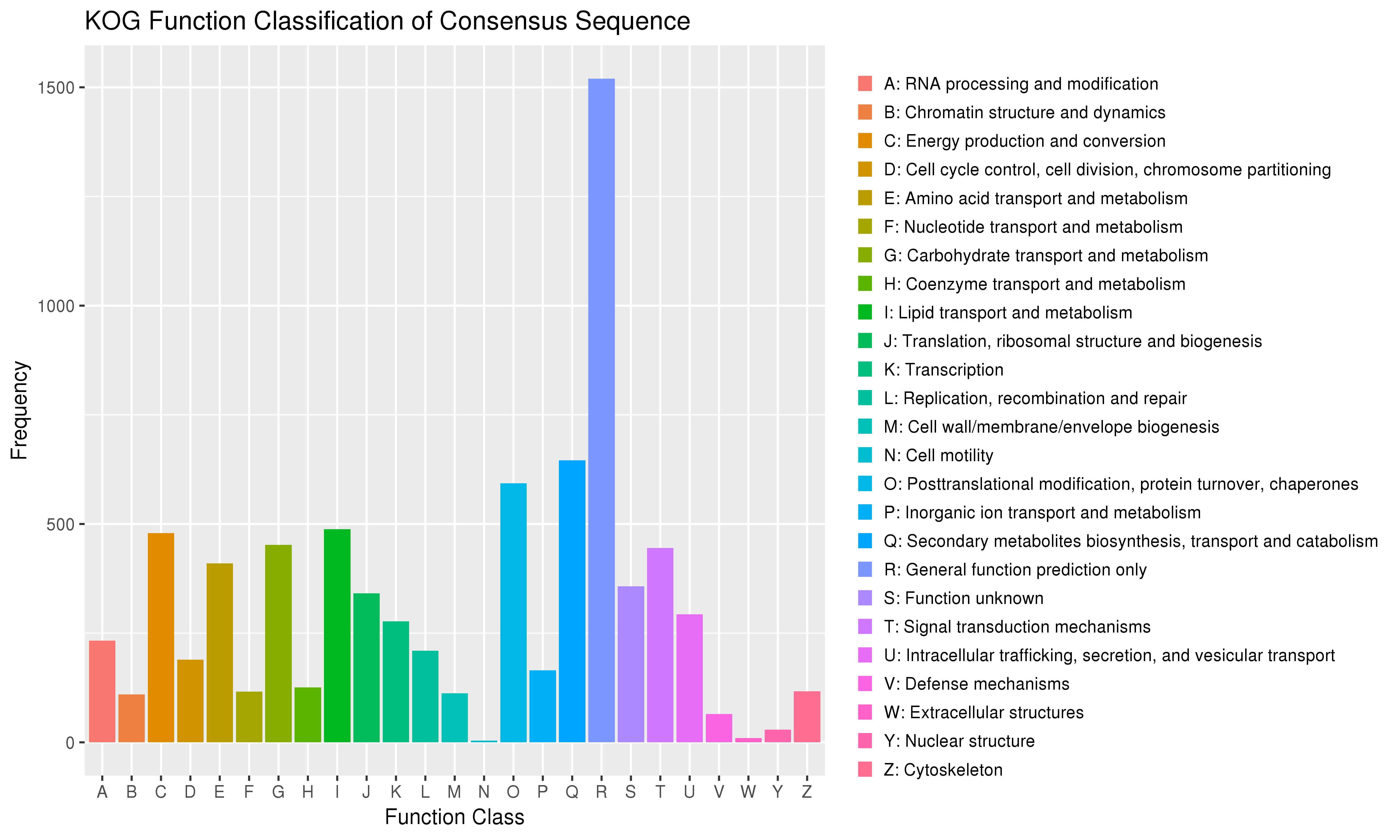

Supplement: Supplementary file 1 [file plants-14-02960-s001.zip › Figure_S2_KOG.png]

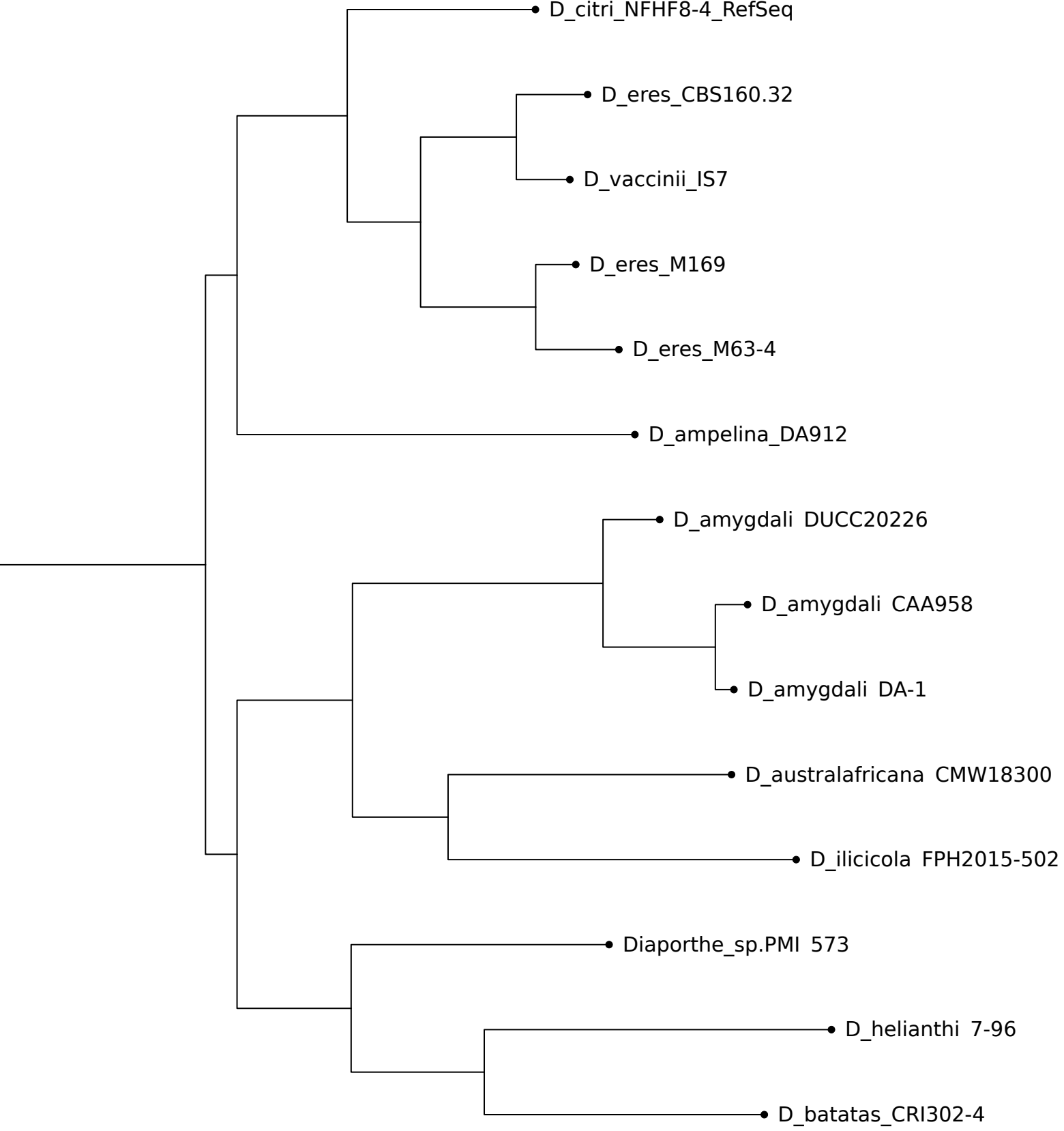

0.02

Supplement: Supplementary file 1 [file plants-14-02960-s001.zip › Figure_S3_speciesTree.pdf]

B)

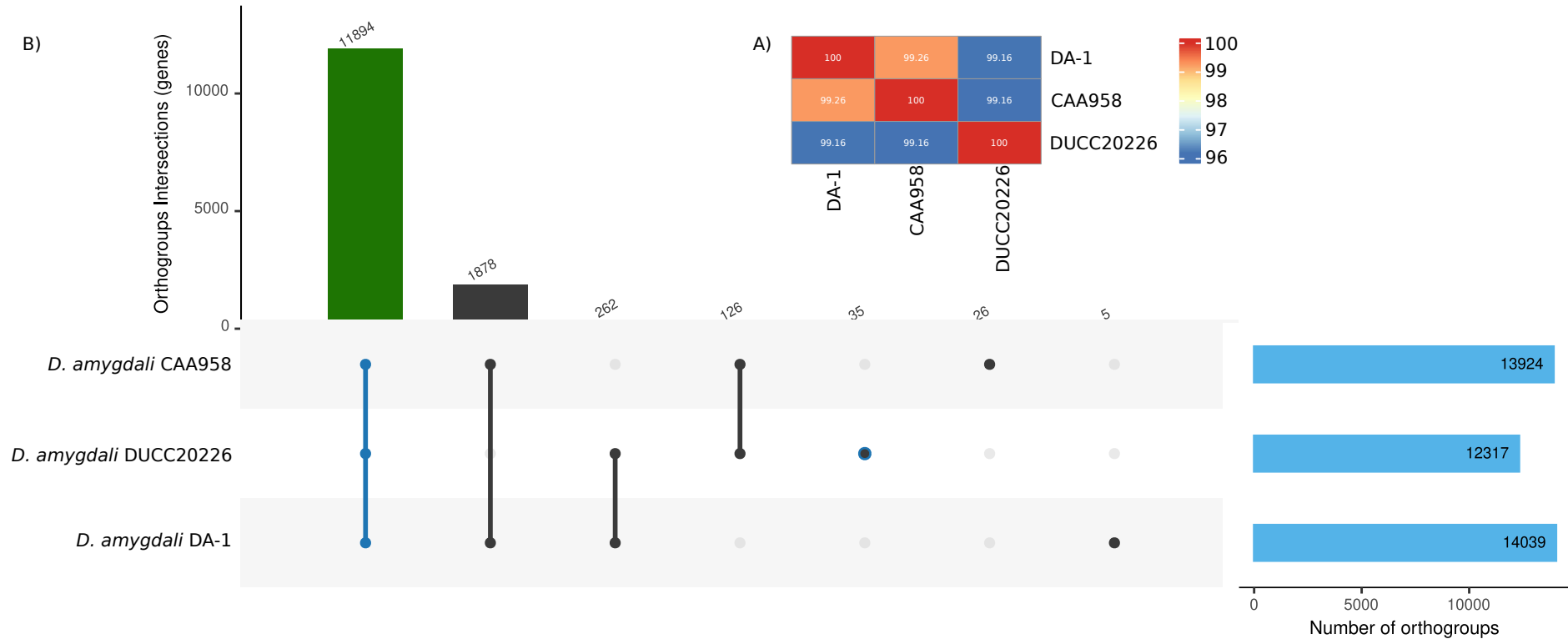

Supplement: Supplementary file 1 [file plants-14-02960-s001.zip › Figure_S4.pdf]
